# Survival in desert: Extreme water adaptations and bioinspired structural designs

**DOI:** 10.1016/j.isci.2022.105819

**Published:** 2022-12-16

**Authors:** Guandi He, Chengqi Zhang, Zhichao Dong

**Affiliations:** 1 CAS Key Laboratory of Bio-inspired Materials and Interfacial Sciences, Technical Institute of Physics and Chemistry Chinese Academy of Sciences, Beijing 100190, China; 2 School of Future Technology, University of Chinese Academy of Sciences, Beijing 100049, China; 3 Key Laboratory of Bio-Inspired Smart Interfacial Science and Technology of Ministry of Education, School of Chemistry, Beihang University, Beijing 100191, China

**Keywords:** earth sciences, environmental management

## Abstract

Deserts are the driest places in the world, desert creatures have evolved special adaptations to survive in this extreme water shortage environment. The collection and transport of condensed water have been of particular interest regarding the potential transfer of the underlying mechanisms to technical applications. In this review, the mechanisms of water capture and transport were first summarized. Secondly, an introduction of four typical desert creatures including cactus, desert beetles, lizards, and snakes which have special adaptations to manage water was elaborated. Thirdly, the recent progress of biomimetic water-collecting structures including cactus, desert beetles, and lizards inspired designs and the influence of overflow on water collection was demonstrated. Finally, the conclusions were drawn, and future issues were pointed out. The present study will further promote research on bioinspired water management strategies.

## Graphical abstract

**Figure:**
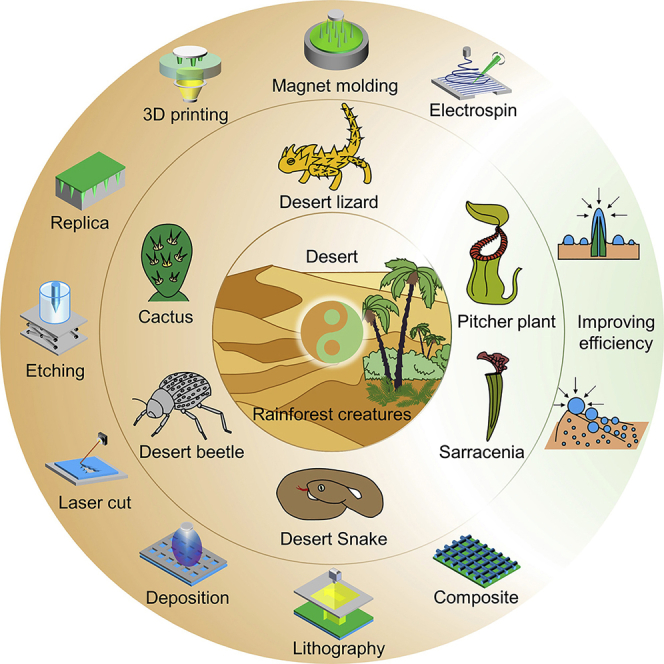

## Introduction

Freshwater is the most indispensable natural resource for life on earth. Since March 22, 1993, World Water Day has become an annual United Nations day that raises awareness of the sustainable management of freshwater.^1^ Although water covers 70% of the earth’s total surface area, freshwater makes up only 2.5%, and most of that is trapped in the form of glaciers and snow.^2^ Available freshwater is only 0.76% in groundwater, 0.001% in the atmosphere, and 0.007% in lakes and rivers.^2^ The imbalance between demanding and available freshwater is worsening in recent years due to environmental pollution, population growth, and economic development.^3^ Over half of the global population is facing severe freshwater scarcity,^4^ and over 2.4 billion people are plagued by the lack of safe water.^5^ To address this issue, researchers focused on finding sustainable sources of freshwater by harvesting from atmospheric fog, purifying from saline water (desalination), and recycling from wastewater.^6^^,^^7^^,^^8^^,^^9^^,^^10^^,^^11^

**Figure 1:**
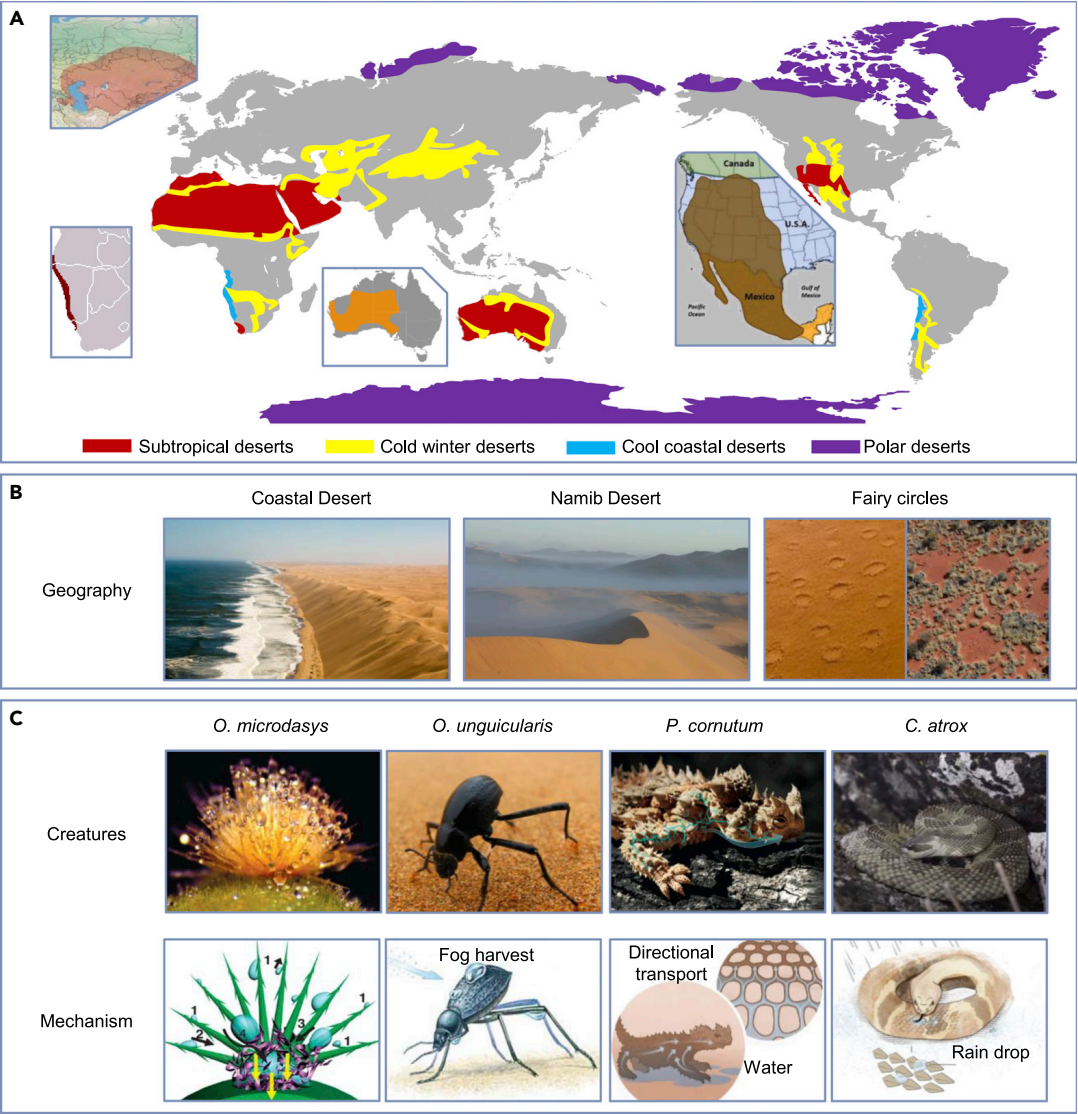
Geographical attributes of deserts and water shortage adaptations of desert creatures (A) Geographical distribution of subtropical deserts, cold winter deserts, coastal deserts, and polar deserts. Inserts show typical habitats of several desert creatures. (B) Images of coastal desert, *Namib Desert*, and fairy circles. Adapted with permission from www.BBC.com, copyright Kate Schoenbach, and Kevin Sanders; and ref.,^14^ copyright 2020 Ecological Society of America. (C) Desert creatures and their special water management adaptations including: cactus *O. microdasys*, desert beetle *O. unguicularis*, desert lizard *P. cornutum*, and desert sanke *C. atrox*. Adapted with permission from ref.,^15^ copyright 2014 American Chemical Society; ref.,^16^ copyright 2012 Springer Nature; www.flickr.com, copyright James Anderson; www.rainharvest.co.za, copyright 2022 Rain Harvest; www.x-bionic.co.uk, copyright 2022 X-Bionic; www.jw.org, copyright 2022 Watch Tower Bible and Tract Society of Pennsylvania; and ref.,^17^ copyright 2019 American Chemical Society.

The word “desert” originated as an ancient Egyptian hieroglyph pronounced *tesert*, which means a place that was abandoned.^12^ Botanically, deserts could be defined as areas with few rainfalls, and with sparse and special vegetation of particular features which allow them to endure harsh environments.^13^ The world’s deserts occupy almost one-quarter of the Earth’s land surface.^12^ They can be divided into four categories (Figure 1A), which include subtropical deserts, cold winter deserts, coastal deserts, and polar deserts.^18^ The subtropical deserts are the hottest, with dry landscapes and rapid evaporation. The coastal deserts are located at the same latitude as the subtropical desert, with a much lower average temperature due to the cold offshore ocean currents. Cold winter deserts are characterized by large temperature differences between seasons, from 38 °C (100.4°F) in summer to 12° C (53.6°F) in winter. Polar regions are also considered deserts because almost all the water in these regions is locked in the form of ice.

Despite the little rainfall in deserts, creatures are not rare, especially in the coastal deserts. Among the coastal deserts, the *Namib Desert* is well known for the spectacular swirling fog around high sand dunes (Figure 1B). It has drawn remarkable scientific attention since it is home to a number of unusual species of plants and animals that are found nowhere else in the world. The eastern edge of the *Namib Desert* is home to a mysterious phenomenon called “fairy circles.”^19^ Fairy circles are large, conspicuous, circular patches devoid of vegetation in the center but with perennial grasses at the margin.^20^ These patches have varying diameters from a few meters to more than 20 m and occur in millions along a narrow belt at the eastern margin of the *Namib Desert*, running from mid-Angola to northwestern South Africa.^20^^,^^21^ For years, fairy circles were thought to exist only in the *Namib Desert* but they were later found 10,000 km away in the remote outback of Australia with the same spatial structure.^22^ Despite the long distance between these two places, the coexistence of fairy circles reminds us of another similarity between them, the adaptation of creatures to obtain and manage available water from sources other than rainfall.

In fact, desert creatures all over the world have evolved special adaptations to survive in the extreme water shortage environment. And utilizing surface structures is one of the major strategies to achieve water collection (Figure 1C). Examples of certain species include: Cactus in the subtropical deserts that have conical spines with a gradient in width to reduce water evaporation and collect water from fog^16^^,^^23^; Desert beetles in the coastal deserts that collect water using their backs with bumpy arrays consisting of hydrophilic peaks and hydrophobic troughs^24^^,^^25^; Desert lizards in the subtropical desert that utilize semi-open surface capillary channel arrays to transport water to the mouth^26^^,^^27^; Desert snakes in the cold winter desert that have dorsal scales with micro-nano structures to pin the impacting water droplets.^17^^,^^28^ These water-collecting and water-handling strategies have been of particular interest regarding the potential transfer of the underlying mechanisms to technical applications, *e.g.* for fog collection,^25^^,^^29^ condensation,^30^ and water transportation.^6^

Bioinspired fog harvesting is a hot topic in biomimetic research and crucial for the sustainable development goal 6 (SDG 6).^31^ Several reviews have summarized the water collection mechanisms in several desert plants or rainforest plants and the bio-inspired structures.^32^^,^^33^^,^^34^^,^^35^ To understand the idea or wisdom from nature creatures, besides these structures, we need to link the natural surroundings, biological adaptations and structures (with variations) in a logical manner. Considering the importance of the underlying rationale, the mechanism of water capture and water transport on solid surfaces was first summarized. Secondly, an introduction of four typical creatures, cactus, desert beetles, lizards, and snakes, which have special adaptations to survive in their habitat was given. Thirdly, the recent progress of biomimetic water-collecting structures and overflow control for water collection were demonstrated. Finally, the conclusions were drawn, and future issues were pointed out.

## Water collection mechanism in desert

### Fog capture

Fog capture is one of the major water collection strategies in desert environments. The freshwater droplets, presented in fog, impact, and condensate on the substrate. The fog collector cannot collect all the liquid water contained in the fog due to the aerodynamic and impact dynamics.^36^^,^^37^ Thus, the overall collection efficiency (*η*) can be expressed as a function of the aerodynamic collection efficiency (*η*_a_) and the subsequent deposition efficiency (*η*_d_). It can be calculated as:

$$\eta =\eta_{a}\cdot \eta_{d}\;,$$

**Figure 2:**
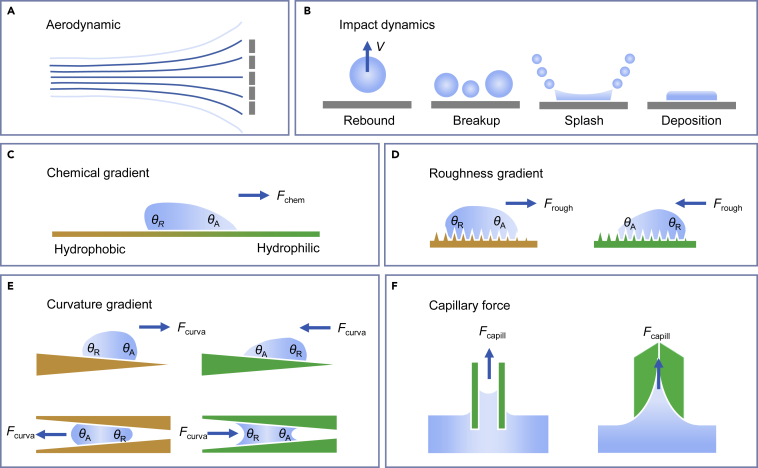
Schematic of water collection mechanism (A) Illustration of air streamlines. The dark blue lines represent effective fog flow, while the bright blue lines represent the diverted fog flow. (B) Different impact outcomes including rebound, breakup, splash, and deposition. (C–F) Water transportation mechanisms. The driving forces for water transport include chemical gradient (C), roughness gradient (D), curvature gradient (E), and capillary force (F). Yellow color means hydrophobic and green color means hydrophilic in all these figures.

The screen-type collector made of mesh is the most common device at present and is taken as the simplified model for discussing the overall collection efficiency (*η*) in detail. As shown in Figure 2A, Part of the flow passes through the mesh and the rest is diverted around it. By treating the flow as the superposition of a flow that passes around a solid screen and a flow that is forced to pass only through the mesh, the aerodynamic collection efficiency *η*_a_ can be expressed as:

$$\eta_{a}=\frac{SC}{1+\sqrt{C_{0}∕C_{d}}}\;,$$

where *SC* is the shading coefficient representing the collector’s area which is capable of capturing droplets, *C*_0_ is the pressure drop coefficient of the device, *C*_d_ is the drag coefficient and corresponds to a nonpermeable screen in this model. It was found large aspect ratio and concave shape could induce a larger drag coefficient (*C*_d_),^38^^,^^39^ which can lead to higher aerodynamic collection efficiency.^36^ The pressure drop coefficient (*C*_0_) depends on *SC* of the fog collector. For most of the mesh collectors, the materials used are plastic, and the calculation of *C*_0_ can be simplified to^36^:

$$C_{0}=k_{Re}\left[1.3SC+{\left(\frac{SC}{1-SC}\right)}^{2}\right],$$

where k_Re_ is an empirical correction factor, which is related to the diameter of the fibers of the mesh.

The deposition efficiency (*η*_d_) quantifies the fraction of fog droplets that are actually deposited to the population initially headed toward the solid wires.^40^ As shown in Figure 2B, different outcomes including rebound, breakup, splash, and deposition can occur after the droplet impacts the surface.^41^ Langmuir developed an empirical correlation for the deposition of small particles on an infinitely long cylinder using numerical calculations.^42^ This correlation is suitable for the mesh collectors to derive the subsequent deposition efficiency *η*_d_ as:

$$\eta_{d}=\frac{St}{St+\pi /2}\;,$$

where *St* is the Stokes number, which is the ratio of the particle’s momentum response time to the flow-field timescale. The deposition efficiency (*η*_d_) is directly related to the Stokes number. Large Stokes numbers (*St* ≫ 1) lead to higher rates of droplet interception and a higher fog collection efficiency.

The resultant overall collection efficiency from the two contributions can therefore be expressed in terms of the shade coefficient of the mesh and the Stokes number of the droplet as:

$$\eta =\eta_{a}\cdot \eta_{d}=\left[\frac{SC}{1+\sqrt{C_{0}∕C_{d}}}\right]\left[\frac{St}{St+\pi /2}\right],$$

### Water transport

The collected water droplets from fog need to be transported timely to ensure sustainable fog capture. On the surface with chemical gradients (Figure 2C), liquids tend to move to areas with higher surface energy, producing a force *F* to drive the collected water drops to move directionally on the surface^29^:

$$F=\int_{l_{i}}^{l_{j}}\gamma \left(\cos\;\theta_{A}-\cos\;\theta_{R}\right)dl,$$

where *θ*_A_ and *θ*_R_ are the advancing and receding contact angles of droplets on the gradient surface, respectively, and d*l* is the integral variable along the length from two regions with different wettability.

As shown in Figure 2D, the surface-free energy gradient attributes to not only the chemical difference of materials but also the surface roughness.^16^^,^^29^ The roughness can be described using Wenzel’s equation:

$$\cos\;\theta_{w}=r\cdot \cos\;\theta ,$$

where *r* is the roughness factor defined as the ratio of the actual surface area to the geometric projected area of a rough surface, and *θ* and *θ*_w_ are the intrinsic and apparent contact angles, respectively. The roughness gradient generates a gradient of wettability, which indicates the surface has a gradient of surface-free energy.^43^^,^^44^^,^^45^ Figure 2E shows that the difference in curvature can generate a Laplace pressure difference (Δ*P*) between different sides of the drop to induce directional transport:^29^

$$\Delta P=-\int_{R_{1}}^{R_{2}}\frac{2\gamma }{{\left(R+R_{0}\right)}^{2}}\sin\;\alpha dz,$$

where *R* is the local radius (*R*_1_ and *R*_2_ are the local radii at two opposite sides of the drop), γ is the surface tension of water, *R*_0_ is the drop radius, *α* is the half-apex angle of the conical spine, and d*z* is the incremental radius.

In addition to dropwise motion, continuous water motion can be triggered by capillary force (Figure 2F). For example, *Moloch horridus* use their semi-tubular skin texture to transport water and import water into their mouths by the capillary rise.^46^^,^^47^ The physical mechanism underlying the capillary process combines the interactions of the surface tension force, viscous drag force, and gravity.^48^ The equation for cylindrical tube can be derived from the classic Stokes Equation as:

$$\rho \left[h\ddot{h}+{\left(\dot{h}\right)}^{2}\right]=\frac{2}{r^{2}}\gamma \;\cos\;\theta -\frac{8}{r^{2}}\eta h\dot{h}-\rho gh,$$

where *ρ* is the liquid density, *η* is the liquid viscosity, *γ* is the liquid surface tension, *r* is the capillary radius, *g* is the gravity acceleration, *h* is the capillary rise height, $\dot{h}$ is the first-order derivative of height *h*, and $\ddot{h}$ is the second-order derivative of height *h*.

Fog collection is one of the most common methods utilized by desert creatures to acquire freshwater. In the next Section, we will review four typical desert creatures, cactus, desert beetles, lizards, and snakes, which have special adaptations to manage water according to their living desert types.

## The water management adaptations of desert creatures

### Cactus

**Figure 3:**
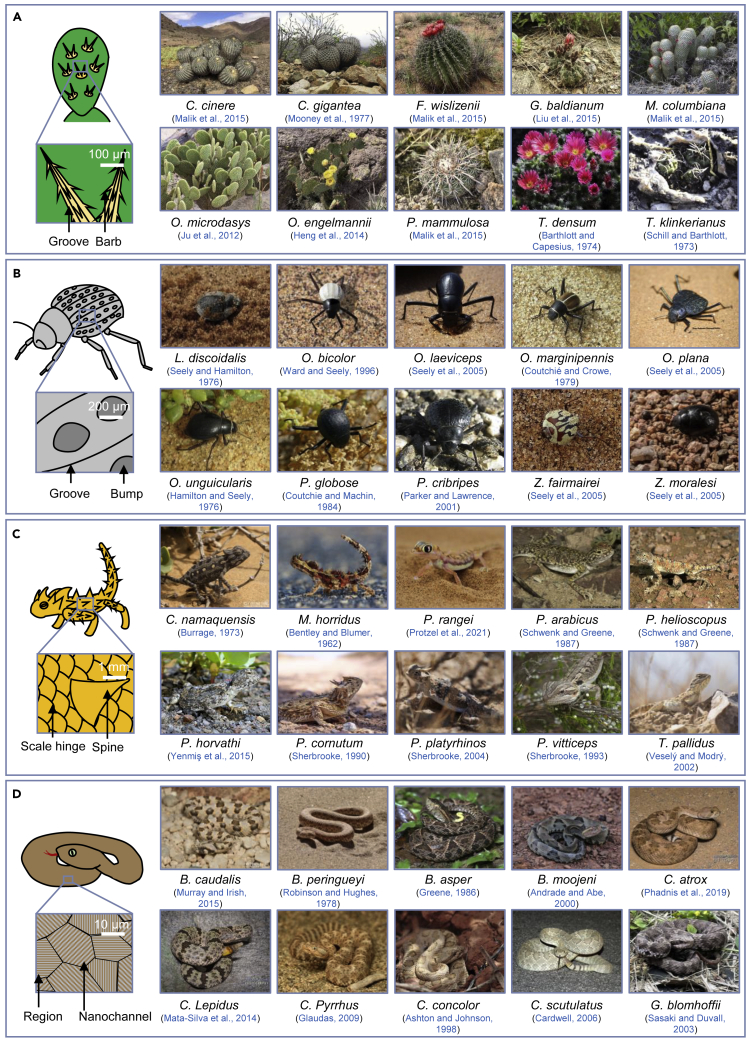
Water harvesting adaptations and certain gifted species (A) Adaptations of cactus including groove and barb and the water harvesting cactus species. Adapted with permission from www.inaturalist.org, copyright Stefan, Juan de los Zorros, CK Kelly, Pablo Demaio, Manuel Ortiz, Carlos Schmidtutz, katkat93, Santiago, Christer T Johansson, and Andrés Zapata. (B) Adaptations of *Namib Desert* beetle including groove and bump and the water harvesting *Namib Desert* beetle species. Adapted with permission from ref. ^14^, copyright 2020 Ecological Society of America; https://pbase.com/, copyright Marielou Dhumez; www.flickr.com, copyright James Anderson; www.inaturalist.org, copyright Martin Weigand, José Antonio Pascual Trillo, and Robert Taylor; and www.margygreen.com, copyright Margy Green. (C) Adaptations of desert lizards including scale hinge and spine and the water harvesting desert lizard species. Adapted with permission from www.inaturalist.org, copyright Tyrone Ping, John Sullivan, wildchroma, Todd Pierson, Roberto Sindaco, highsinger, Tony Iwane, and Mark Hura, Roberto Sindaco; and www.wondersofcoldblood.com, copyright Thor Håkonsen. (D) Adaptations of desert snakes including region and nanochannel and the water harvesting desert lizard species. Adapted with permission from www.tyroneping.co.za, copyright Tyrone Ping; www.inaturalist.org, copyright charleyhesse, Jake Scott, Jessica dos Anjos, Zach Lim, Ethan Gosnell, matthew gruen, and Gregory Mihaich; and www.mjacobi.com, copyright Michael Jacobi.

Many members of the Cactaceae family can survive in highly arid deserts. They are mainly distributed in subtropical deserts in southern China, Southeast Asia, South America, and Africa.^49^^,^^50^^,^^51^^,^^52^ Cactus leaves have evolved into needles after millions of years of evolution, which reduces the surface area to limit water transpiration and enable better water storage.^53^^,^^54^^,^^55^ Besides the minimization of water loss, some species (Figure 3A) utilize the multifunctional spines to collect freshwater from fog as a supplementary water source.^16^^,^^56^^,^^57^^,^^58^^,^^59^^,^^60^^,^^61^^,^^62^ Water drops can be absorbed into the stem, especially in foggy areas.^63^^,^^64^ Subsequent to the absorption, water can be reserved in the mucilage cells in the stem, containing polysaccharides with a high affinity to water to reduce the evaporative loss.^65^

**Figure 4:**
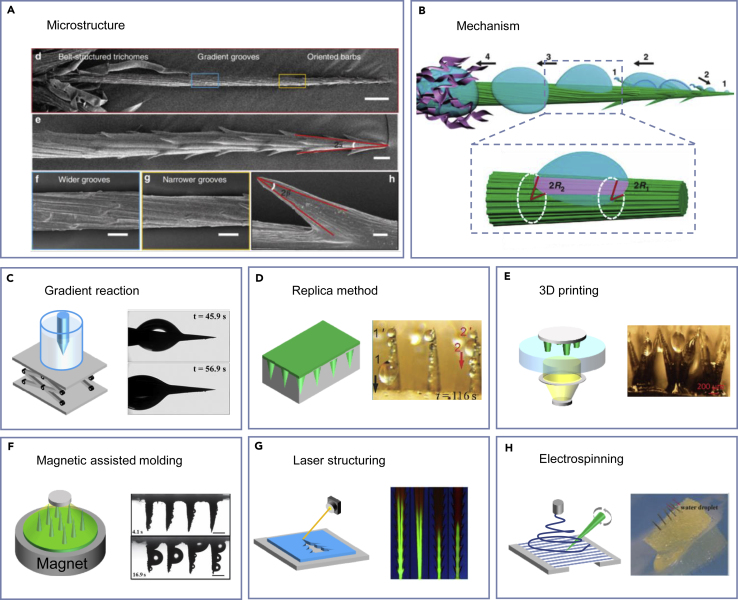
Mechanism of cactus-inspired water management and the preparation processes (A) Magnified SEM images of the hierarchical structure of the cactus, including trichomes, grooves, and barbs. Adapted with permission from ref.,^16^ copyright 2012 Springer Nature. (B) Schematical overview of the cactus fog collection by deposition, collection, transportation, and absorption. Adapted with permission from ref.,^16^ copyright 2012 Springer Nature. (C–H) There are six preparation processes of cactus-inspired water collection devices: (C) Gradient reaction. (D) Replica method. (E) 3D printing. (F) Magnetic assisted molding. (G) Laser structuring. (H) Electrospinning. Adapted with permission from ref.,^23^ copyright 2013 Wiley-VCH; ref.,^66^ copyright 2014 Wiley-VCH; ref.,^67^ copyright 2019 Wiley-VCH; ref.,^68^ copyright 2014 Wiley-VCH; ref.,^69^ copyright 2020 American Chemical Society; and ref.,^70^ copyright 2015 Wiley-VCH.

One example of a fog-harvesting cactus is *Opuntia microdasys* originating from the Chihuahua Desert, which has an integrated multifunctional system that facilitates efficient fog collection.^16^ This unique system comprises well-distributed clusters of conical spines on the cactus stem. According to their surface structural features, each cluster contains three integrated parts with different roles in the fog collection process (Figure 3, Figure 4 and 4A). These include (i) oriented conical barbs on the tip, (ii) gradient grooves in the middle, and (iii) belt-structured trichomes on the base. The condensation primarily happens on the barb and the spine, with water drops moving directionally toward the base. As the deposition proceeds and the water drops coalesce, these drops increase in size and leave the tip side of the spine (Figure 4B). The coalescent drops are then further transported along the gradient grooves and absorbed through the trichomes at the base of the spines. In addition to the clusters of spines and trichomes, it is found that the intersite of the clusters on the cactus stem is covered with densely distributed cones, contributing to the overall fog collection.^66^

The integration of the multiple functions within the spines and the trichomes, including water deposition, collection, transportation, and absorption in the cactus, facilitates an efficient fog collection system. The conical shape of the spines and barb results in a larger radius near the base than the tip. This difference in radius generates a Laplace pressure difference, which drives droplets to the side with a larger radius.^71^ In addition to the gradient of the Laplace pressure, the microgrooves on the cactus spines have a gradient in width along the spine, decreasing from the base to the tip. This gradient in width leads to a difference in roughness, which generates a wettability gradient. Since the surface of the cactus spines is covered with vegetable wax, the tip is rougher and more hydrophobic, while the base is less rough and less hydrophobic.^63^ This difference drives the water drops collected on the tip directionally toward the base. Besides, the oriented barbs reduce the drop’s ability to spread or move toward the tip side with the barbs, facilitating movement toward the base side lacking barbs.^16^ The aligned grooves can also generate an anisotropic contact angle hysteresis in the direction parallel or perpendicular to the grooves, enhancing the directional movement of the water drops along the grooves on the barbs and spines.^72^^,^^73^ Water droplets can move more readily in the parallel direction to the aligned groove structure than in other directions.^74^ The droplet has a continuous three-phase contact line along the groove, which reduces the energy barrier and is therefore conducive to the diffusion and movement of the droplet.^75^

### Desert beetle

The *Namib Desert* is coastal desert and comprises a long, narrow desert situated in southwestern Africa. The cold Benguela coastal current suppresses rainfall over the desert and leads to fog.^76^ Most of the water collected in the coastal area of the *Namib Desert* is advective fog rather than normal precipitation rainfall, which is a peculiar feature of the desert.^77^ Fog brings water in the form of tiny droplets that can deposit up to a liter of water per square meter on the mesh of an artificial fog screen during a day in the *Namib Desert*.^78^ There are other coastal foggy deserts, for example, the *Atacama*, *Baja California*, *Omani*, and *Yemeni Deserts*. However, no other coastal foggy desert has a combination of the cool coastal climate, large sand dune mass, and gently rising land from the coast of the *Namib Desert*.^14^

Under such a unique environment, numerous Darkling beetles found in this desert have adopted different mechanisms to utilize fog for survival (Figure 3B).^24^^,^^25^^,^^79^^,^^80^^,^^81^^,^^82^ Some construct sand trenches to catch the fog, while others utilize their body surface as a fog water collector.^24^^,^^81^ In the former case, trenches are constructed perpendicular to fog winds, and moisture concentrates along the trench ridges, where beetles return along and extract water. In the latter case, a typical stance is adopted, facing the body toward the wind, and the fog water is collected on their elytra and flows down to their mouth to be swallowed. Fog collection becomes critical when rainfall is absent over prolonged periods. Long-term studies of the population density of Darkling beetles in the *Namib Desert* show that the fog-collecting beetles persisted during dry periods, whereas the others that lack this adaptation disappear or decline to less than 1% of their mean abundance.^80^

In 1976, Hamilton and co-workers reported field observations of direct water uptake from the fog of a *Namib Desert* beetle, *Onymacris unguicularis*.^24^ A steady flow of collected water was later observed on *O. unguicularis*.^83^ In 2001, a water-capturing mechanism by which fog water forms into large droplets on a beaded surface was described in the study of the elytra of beetles *Stenocara* sp.^25^ The structures behind this were believed to be an array of non-waxy hydrophilic bumps 0.5-1.5 mm apart and about 0.5 mm in diameter surrounded by a wax-coated hydrophobic region. Water carried by fog can accumulate in the hydrophilic part and form droplets that grow in size. The droplet can eventually reach a critical size when the capillary force that attaches the droplet to the surface is surpassed, and the droplet detaches and rolls down. However, this water-capturing mechanism was challenged by Hamilton et al. for its applicability to collecting fog water in nature.^84^ They also reidentified the beetle in the study as *Physasterna cribripes* rather than a *Stenocara* sp.^84^ Following reports found *P. cribripes* could not exhibit fog-basking behavior, and entire bumpy surfaces are homogeneously covered with hydrophobic wax.^83^ A higher droplet nucleation rate is found in valleys between the bumps, where dew forms primarily.^85^

Despite the debate mentioned above, the hetero-wettability pattern has been proven to facilitate fog collection by many hetero-wettability patterned artificial surfaces. With most previous studies focusing on surface chemistry, morphology including bumps and grooves has been overlooked. The bumps on the beetle’s back could facilitate condensation by focusing vapor diffusion flux at the apex.^86^ Grooves reduce the energy barrier of water movement,^75^ which could be essential for capturing the water droplets or conveying them to the beetles’ mouths.

### Desert lizards

As shown in Figure 3C, a number of desert lizard species, mostly inhabit subtropical deserts, have a spectacular ability to harvest environmental moisture using their body surface.^26^^,^^87^^,^^88^^,^^89^^,^^90^^,^^91^^,^^92^^,^^93^^,^^94^ Two typical examples include the Australian thorny devil (Agamidae: *M. horridus*) and Texas horned lizard (Iguanidae: *Phrynosoma cornutum*).^26^^,^^91^ While *M. horridus* lives in the desert regions of Western and South Australia, *P. cornutum* occupies a variety of open desert and grassland habitats in North America.^26^^,^^95^ Despite the geographical isolation, convergently evolution leads to the co-occurrence of the specific features to facilitate water capture and transport for drinking.^47^ Moisture harvesting consists of two elements: a specific behavior combined with special body postures, and a particular morphology of the integument allowing the collection and transport of water toward the mouth.^27^ The stereotypic body posture of most moisture-harvesting lizards includes similar stretching of the hind limbs and lowering of the head.^89^^,^^91^^,^^94^ Gravity appears to be utilized to support the functionality of skin microstructures and channels. Particular morphology of the integument seems to be a more common character for moisture-harvesting lizards. Special surface microstructures of the outer layer of the epidermis and capillary channels in between the scales allow the lizards to collect water into their skin capillaries and transport it to their mouth for drinking.^27^^,^^47^ The integument of lizards consists of an outer beta-keratin layer, an inner alpha-keratin layer, and a mesos layer separating the two former layers.^96^ The outer β-layer is covered by the corneous protective Oberhäutchen.^47^ The hydrophilicity is linked to honeycomb-shaped microstructures on the Oberhäutchen, which keeps a film of water after pre-wetting.^27^^,^^47^ Typical dimensions of these microstructures are 10-30 μm in diameter and 1-5 μm in depth.^27^ Because the mesos layer contains extracellular lipids, it is responsible for the water impermeability of the lizard’s integument and hence protects the lizard from desiccation.^47^

Water is transported in the channels between the scales rather than simply spread over the skin surface, as most of the scale surfaces are not covered by water during transport.^97^ Transportation in channels avoids wetting much of the body surface and hence losing volume by evaporation from a larger area.^27^^,^^90^ The amount of water accumulation in cavities of the integumental surface of *M. horridus* is 9.19 mg cm^−2^, which is higher than *P. cornutum* with a value of 5.9 mg cm^−2^.^92^^,^^98^ The channels have globular protrusions that form narrow subchannels of up to 50 μm depth and their surface is covered with an Oberhäutchen surface structure.^47^^,^^99^ The volume of the sub-channel structure is estimated to be about 50% of the total channel volume.^97^

Capillary water transportation needs sufficient water to fill the capillary channels, so that capillary water finally reaches the mouth for ingestion.^98^ The volume of water held in the cutaneous capillary system is about 3-4% of the body mass.^99^^,^^100^ Two structural modifications have been found that most likely enable the lizards to drink even smaller amounts of water than are sufficient for the complete filling of the channels.^101^ First, in the hierarchical channel structure, large cavities can quickly absorb water into their high-volume channel system, whereas sub-capillary structures yield an extension of the transport distance.^97^ Second, a directional water transport toward the mouth has been found in *P. cornutum*, and results from a combination of asymmetric channel geometry and interconnection network structure.^46^ Narrowing of single channels between two neighboring scales enables local directional liquid transport, whereas specific interconnections help the water to transport toward the mouth.^46^

### Desert snakes

Desert snakes that mostly inhabit cold winter deserts can also use their body to collect rain droplets and the surface properties of their dorsal scales play a key role in this process. In the absence of freestanding water sources, various species of desert snakes (Figure 3D) have been reported to use their bodies to harvest rain for drinking.^17^^,^^28^^,^^102^^,^^103^^,^^104^^,^^105^^,^^106^^,^^107^^,^^108^^,^^109^ These snakes are reported to display a stereotyped rain-harvesting posture (Figure 3D), considerably flattening their bodies and forming a tight coil, presumably to enhance the collection of rain droplets. As the rain droplets accumulate and coalesce on the dorsal scales, the snake proceeds to drink the water from various areas of its body. The functional significance of the water-collecting behavior is suggested to be related to the acquisition of water from short rainfalls.

One of the water-harvesting species, the Western Diamond-backed Rattlesnake (*Crotalus atrox*) from southern *Arizona*, has been observed to emerge from rock-structured dens even during late winter to harvest rain, sleet, and snow.^110^ It was demonstrated that the nanotexture and wettability of the skin of *C. atrox* aid in rain droplet capture for drinking.^17^ Compared with species that were not known to show rain-harvesting behavior, the scales of *C. atrox* exhibited a higher water contact angle and have a dense labyrinth-like nanotexture. When interacting with rain, this shallow nanotexture firmly captures water droplets by pinning the triple-phase contact line.

Two other surface structures, keels and region boundaries, may also play important roles in water harvesting and require further investigation. Keels are macroscale protrusions of the scales and run at the center along its length. It can potentially help to split some of the impacting droplets and reduce the Weber number. Region boundaries are thin and mostly straight boundaries that can separate the nanotexture into four-to five-sided regions that measure 30-50 μm across. These microscale boundaries may also help with pinning the liquid droplet.

Inspired by the water management adaptations of the desert creatures, the biomimetic water collection surfaces, and water collection facilitates were designed and manufactured extensively. In the next Section, we will review the recent progress in the manufacturing methods of the biomimetic water-collecting structures and demonstrate the influence of overflow on water collection.

## Bioinspired surface structure designs

### Cactus-inspired designs

#### Gradient reaction

A gradient electrochemical reaction was proposed to make the cactus-inspired wires. This method enables the gradient by repeatedly raising and lowering the electrolyte container or the metal wire to endow gradient reaction time (Figure 4C). With this method, Ju et al. performed gradient corrosion and subsequently chemical modification to prepare conical copper wires with increasing wettability from tip to base.^23^ The hydrophobic tip of the conical copper wire ensures a quick collection of water drops. The gradient Laplace pressure from the conical shape and the gradient of wettability arising from the gradient chemical modification guarantees fast transportation of the drops. Zhou et al. designed a micro/nanostructured conical spine and Janus membrane integrative system by gradient electrochemical reaction.^111^ In this strategy, aluminum wire is made into the conical spine by gradient electrochemical reaction and then covered with rough micro and nanostructure by hydrothermal method; Janus membrane with an inside hydrophobic surface and outside hydrophilic surface is further used to control the water collection. Another artificial cactus-inspired structure was achieved with ZnO by adopting a two-step vapor phase method.^58^ The wires had conical shapes mainly due to the spatial variation of reactant concentration. Due to the diameter gradient from top to bottom, the condensate drops on the top tend to be driven to the bottom. The comparatively larger surface areas of the branched wires allow this structure to collect more water than the natural cactus spine.

#### Replica method

As shown in Figure 4D, artificial polydimethylsiloxane (PDMS) cone arrays are prepared using a facile method combining mechanical perforating and template replica technology.^66^ The PDMS cone arrays are fabricated with different arrangements. The one in hexagonal arrangement proved to be more efficient due to more turbulent flow around the staggered cones and the rapid directional movement of water drops along each cone. Using a similar mechanical punching and replica method, cactus spine-like magnetic arrays were fabricated by Peng and co-authors.^112^ Magnetically induced fog harvesting under windless conditions can be achieved by integrating cactus-inspired spine structures and magnetically responsive flexible conical arrays. Fog can be continuously captured and directionally transported from the tip to the base of the spine through periodic vibration driven by the external magnetic field and the Laplace pressure difference arising from the conical shape.

#### 3D printing

As shown in Figure 4E, 3D printing is another effective way to fabricate customized parts with complicated cactus-inspired structures. This method has the merits of easy control and large-scale production. Li et al. reported a 3D-printed water collector with microscale biomimetic branched spines with controllable spine tip angle and wettability to improve the water collection efficiency (Figure 4E).^67^ The 3D-printed hexagonally arranged multibranched spines with hydrophobic nano-coating and optimized tip angle showed apparent advantages over other structured surfaces. However, due to the intrinsic properties of the layered additive building process, obvious stair steps exist, and fabrication time is long due to layer-by-layer curing. Liu et al. tackled the problem by utilizing a custom-made high-resolution micro-continuous liquid interface printing (μCLIP) technology.^113^ In addition to an array of cactus-inspired spines, this fog collector incorporated features of desert grass-inspired longitudinal ridges and *Nepenthes alata* peristome-inspired bottom channels. Environmental fog is captured at the tips, channeled down to the base, and transported through the grooves to the bottom reservoirs. The new collector showed obvious advantages to collecting fog efficiently by involving fog capture and water transportation steps.

#### Magnetically assisted molding

Cao et al. reported another cactus-inspired artificial fog collector with conical micro tips by magnetically assisted molding (Figure 4F).^68^ Conical micro-tips were shaped by applying an external magnetic field to a mixture of PDMS and magnetic particles. After curing, the resultant conical micro-tips exhibited cactus spine-like fog collection ability driven by the Laplace pressure difference. A large-scale cactus-inspired fog collector was subsequently fabricated by integrating conical arrays with a water-absorbable cotton matrix, achieving spontaneous and continuous collection and preservation of water. In another fog collector, the directional magnetic field was likewise applied on magnetic curable fluid to make cactus-inspired conical spines with oriented conical barbs on a superhydrophilic substrate.^114^ This fog collector can achieve spontaneous and continuous deposition, coalescence, transport, and absorption of water. These are mainly attributed to the synergistic effect of the Laplace pressure gradient generated by the conical shape of the spine and micro-barbs, capillary pressure arising from the concave meniscus between the spine and backward barbs, and the wettability of the superhydrophilic substrate.

#### Laser structuring

Originating from traditional paper art, “kirigami” and “origami” have enabled a branch of promising functions. Taking inspiration from cactus spines, Bai et al. present a cactus kirigami made by laser structuring for highly efficient fog harvesting (Figure 4G).^115^ This method simplified the 3D complicated structure to a 2D hollow plate while preserving the ability of directional and continuous droplet delivery. The wax-infused kirigami with an anisotropic shape fulfills efficient capture of fog droplets and rapid refreshing of the surface through directional droplet self-propulsion. Inspired by the conical spine with barbs on the cactus and microchannels on *the Sarracenia* trichome, Wang et al. fabricated a spine with barbs and hierarchical channels by laser structuring.^69^ This structure exhibited fast water transportability and high fog harvesting efficiency. Scaled-up fog collectors are subsequently designed with the basic unit using direct laser structuring and origami techniques, which showed high-efficient fog collection capacity.

#### Electrospinning

As shown in Figure 4H, Bai et al. focused on the hierarchical groove structure of cactus and developed an artificial “cactus spine” by electrospinning and a sacrificial template method.^70^ Composite fibers were electrospun to span across the electrode gap. Then, a micrometer silver needle was rotated along the aligned fibers at a fixed angle to cover the fibers onto their surface. After an imidization treatment, an artificial cactus spine with a hierarchical groove structure could be obtained. The artificial spine showed excellent fog collection and water transportation performance due to the particular groove structure.

### Desert beetle-inspired designs

#### Selective deposition

**Figure 5:**
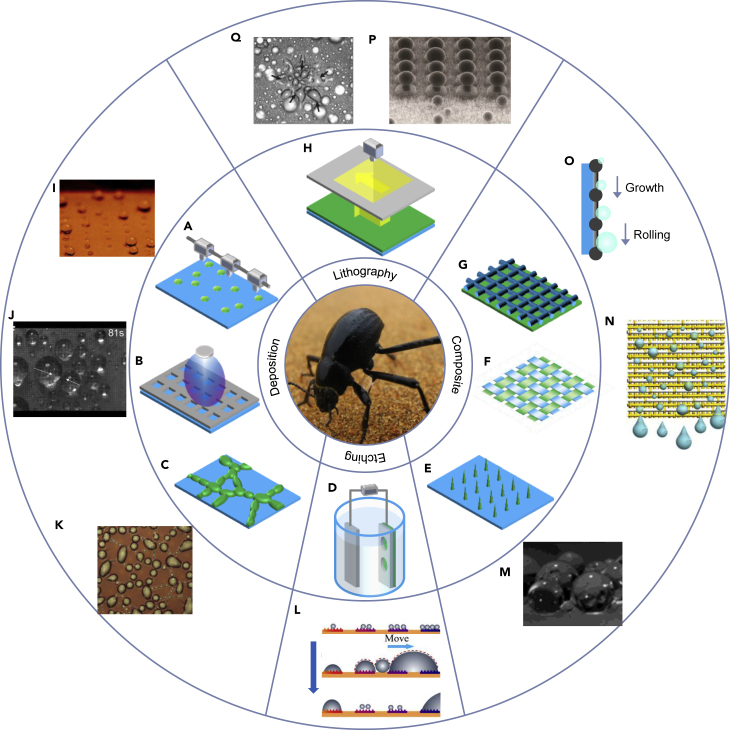
Schematics of four preparation methods of beetle-inspired devices and water harvesting mechanism (A–K) There are four preparation methods categories of beetle-inspired devices: (A–C) Selective deposition. (D) Selective chemical etching. (E–G) Composite with hydrophobic and hydrophilic components. (H) Lithography. (I–Q) Water harvesting mechanism of beetle-inspired devices including: (I–K) Mechanism on selective deposited devices. (L) Mechanism on selective chemical etching devices. (E–G) Mechanism on composite with hydrophobic and hydrophilic components. (H) Mechanism on lithography prepared devices. Adapted with permission from ref.,^116^ copyright 2006 American Chemical Society; ref.,^117^ copyright 2017 Wiley-VCH; ref.,^118^ copyright 2015 American Chemical Society; ref.,^119^ copyright 2019 American Chemical Society; ref.,^120^ copyright 2015 American Chemical Society; ref.,^121^ copyright 2018 American Chemical Society; ref.,^122^ copyright 2015 American Chemical Society; and ref.,^123^ copyright 2014 Wiley-VCH. Middle figure adapted with permission from www.flickr.com, copyright James Anderson.

The selective deposition method can be mainly subdivided into mask-based and mask-free strategies. Inspired by the beetle’s back, a surface with an array of hydrophilic spots on a superhydrophobic surface was created by depositing hydrophilic polyelectrolytes.^116^ As shown in Figure 5I, spraying a mist of water onto the surface leads to nearly perfect spherical water droplets that do not wet the superhydrophobic surface, eventually trapped in the patterned hydrophilic regions. Dorrer et al. reported another deposited surface by fabricating nano-grass structures on a silicon surface, followed by surface patterning with repeated dispensing of polymer solution and evaporation of the solvent.^124^ The surface energy in the hydrophilic regions was carefully controlled to manage the wetting behavior of the drops. Wang et al. reported the deposition of TiO_2_ sol by spraying with a unique raised structure on a superhydrophobic fabric surface.^125^ The TiO_2_ nanosol bumps have light-induced superhydrophilicity, which may provide ideas leading to the development of smart water collection devices. Hou et al. reported an electrospraying method that can enable scalable fabrication of hetero-wettability patterns by spraying a nylon solution on a functionalized aluminum surface (Figure 5A).^126^ The electrospraying technique can rapidly generate nanoscale structures on surfaces of various shapes and conveniently adjust the structural geometry and distribution of the hydrophilic patterns. Another direct one-step and mask-free method of depositing that can enable facile large-scale patterning is inject printing. An aqueous dopamine droplet was directly applied by inkjet printing to superhydrophobic surfaces, followed by the *in situ* formation of poly-dopamine to obtain superhydrophilic micropatterns.^127^

As shown in Figure 5B, the mask-based selective deposition strategies need a pre-designed mask with a pattern, which is transferred to the surface. As reported by Garrod et al., hydrophilic polymers have been deposited through a mask via plasma deposition onto a superhydrophobic polymer substrate.^128^ Yu et al. fabricated Pt nanoparticles-coated hydrophilic patterns on the PDMS-coated superhydrophobic surfaces using a pulsed laser deposition (PLD) approach with masks.^117^ This approach is proved to be a versatile and effective strategy for constructing large-scale superwettable patterned surfaces for water harvesting (Figure 5J). Zhong et al. utilized the effect of bulgy topography and wettability for fog harvesting.^129^ The bulgy topography was achieved by pressing the copper foil with high pressure. The hetero-wettability pattern was fabricated by the selective deposition of TiO_2_ nanoparticles onto the superhydrophobic background using a mask, followed by modification with thiols.

Another deposition method to create patterned water-collecting surfaces was the spontaneous dewetting of polymer bilayer thin films (Figure 5C).^118^^,^^130^ Dewetting is a symmetry-breaking process whereby an unstable liquid film is transformed into a series of isolated droplets driven by unfavored intermolecular forces at the interface between two materials (Figure 5K). This mechanism occurs only when the system is annealed above the glass transition temperature to enable sufficient mobility. Thickett et al. first created a pattern from the spontaneous dewetting of hydrophilic poly(4-vinyl pyridine) from hydrophobic polystyrene to yield surfaces with chemical and topographical contrast.^130^ Wong et al. used the same method and formed isolated poly(2-hydroxypropyl methacrylate) (PHPMA) domains atop polystyrene film.^118^

#### Lithography

As shown in Figure 5H, lithography can likewise fabricate beetle-like microstructures. Bai et al. fabricated a surface with star-shaped hetero-wettability patterns inspired by desert beetles and spider silk.^123^ Superhydrophilicity was first achieved by spin-coating of TiO_2_ slurry. Then the film is treated with a silane to change the wettability to superhydrophobic. Subsequently, circle-shaped or star-shaped photomasks were used to obtain superhydrophilic features via selective exposure to UV light. By integrating a surface energy gradient and Laplace pressure gradient, surfaces with star-shaped wettability patterns can quickly drive tiny water droplets toward more wettable regions to avoid being lost in the wind (Figure 5Q).

As shown in Figure 5P, Hou et al. developed a hybrid surface with high wetting contrast that allows the integration of film-wise and dropwise condensation modes.^122^ SiO_2_ patterns on the silicon wafer were first fabricated using standard photolithography and oxide etching processes. Then, micropillars with SiO_2_ tops were further etched, and nano-grass covering the valleys of the micropillar arrays was also achieved by etching. By confining the hydrophilic patches on the top of the micropillars surrounded by superhydrophobic nano-grass, such a surface accelerated droplet nucleation and depinning.

Wang et al. developed a synthetic surface combining the topography and surface chemistry of desert beetles and cactus.^131^ The silicon bump arrays were first fabricated using standard lithography and etching processes. Then superhydrophobic paint was sprayed on the patterned surface using an airbrush and dried. Finally, the superhydrophobic coating on the top surface of the bump arrays was ground off, while the coating on the sidewall and bottom of the bump was reserved. The synthesized surface exhibited a high nucleation rate and stable dropwise condensation and promoted rapid droplet self-removal.

#### Composite with hydrophobic and hydrophilic components

The first artificial composite with hydrophobic and hydrophilic components was created by Parker et al. following the discovery of the wax-coated and non-waxy regions on the beetle’s back.^25^ By partially embedding the glass spheres into warm wax, more water can be collected compared with a uniformly hydrophobic surface of smooth wax or a uniformly hydrophilic surface of bare glass.

The copper mesh has been extensively discovered to produce bioinspired composites with hetero-wettability components. As shown in Figure 5G, Wang et al. incorporated a modified hydrophobic copper gauze onto the top of a hydrophilic polystyrene flat sheet by thermal pressing method.^132^ The produced hybrid patterned surfaces consisted of polystyrene patches sitting within the holes of the copper gauzes. The low-cost and wide availability of polystyrene and copper gauze allows this method to scale up. Cao et al. constructed a Janus system with hydrophobic copper mesh and hydrophilic cotton absorbent, demonstrating an enhanced efficiency potential for fog harvesting.^133^ The fabrication process of this Janus system was facile, scalable, and cost-efficient. Yin et al. reported another simple, low-cost method to prepare a hybrid hetero-wettability pattern surface with copper mesh.^134^ The surface is constructed by incorporating femtosecond laser-induced polytetrafluoroethylene (PTFE) nanoparticles deposited on copper mesh with a hydrophilic copper sheet. The as-prepared surface exhibited enhanced fog collection efficiency and anti-corrosion properties. Based on copper mesh, Zhang et al. presented a multi-bioinspired patterned fog collector with hydrophilic nanofibrous bumps and a hydrophobic slippery substrate.^135^ The hydrophilic nanofibrous bumps increase the effective fog-collecting area, while the hydrophobic slippery substrate promotes rapid transport of collected water. Hu et al. designed a hybrid membrane with anisotropic wettability and asymmetric micro-topology.^136^ A layer of hydrophobic polymer nanofibers is firstly electrospun onto Cu mesh, followed by anodization to grow Cu(OH)_2_ nanoneedles from the Cu mesh, which can pierce through the polymer nanofiber network. The hybrid membrane provided a cooperative mechanism for fog harvesting due to its special wettability and topology anisotropy, and could collect sufficient fog water for typical irrigation requirements of plants in foggy areas.

Mondal et al. developed an array system of hydrophilic needles thermally connected to a copper heat sink, which was forced through a superhydrophobic polymer film (Figure 5E).^120^ Condensation occurs preferentially on the needle surface due to wettability and temperature differences (Figure 5M), and the droplets roll off the surface after reaching a critical volume. As shown in Figure 5F, Gao et al. introduced a hetero-wettability patterned weft-backed woven fabric surface by a facile weaving method with simple textile equipment.^121^ The hybrid wettable surface was produced by hydrophilic viscose and hydrophobic polypropylene yarns produced by common commercial agents, which relieved the cost, making it convenient for mass production. The water-harvesting fabric achieved a water harvesting rate 59.2 times higher than that of beetles and showed high reusability after ten times of recycling.

#### Selective chemical etching

The electrochemical-etching method has been adopted to create the wettability contrast. Inspired by the wettable patterns of the *Namib Desert* beetle and the wettable gradient of spider silks, Xing et al. propose a fog collection system via one-step anodic oxidation (Figure 5D).^119^ The masked surface cannot be oxidized except for the areas exposed to electrolytes, so wettable patterns are obtained during the anodic oxidation process. Besides, a wettable gradient is formed on the surface due to the current density and oxidation time gradient induced by emptying the electrolyte. The obtained surface can improve the fog droplet capture performance because of hydrophilic patterns and maintain effective water drainage with the hydrophobic substrate during the fog collection process (Figure 5M). Yang et al. reported a twice electrochemical etching process and achieved a hetero-wettability patterned surface with a mask.^137^ Electrochemical-etching and surface chemical modification technologies were first utilized to prepare superhydrophobic substrates. Then, the electrochemical-etching technology was applied with a mask to fabricate superhydrophilic dimples on the prepared superhydrophobic surfaces directly. The fog harvest test shows that a superhydrophobic surface with patterned arrays has a high rate of water collection due to the rapid condensate drainage.

### Lizard-inspired designs

**Figure 6:**
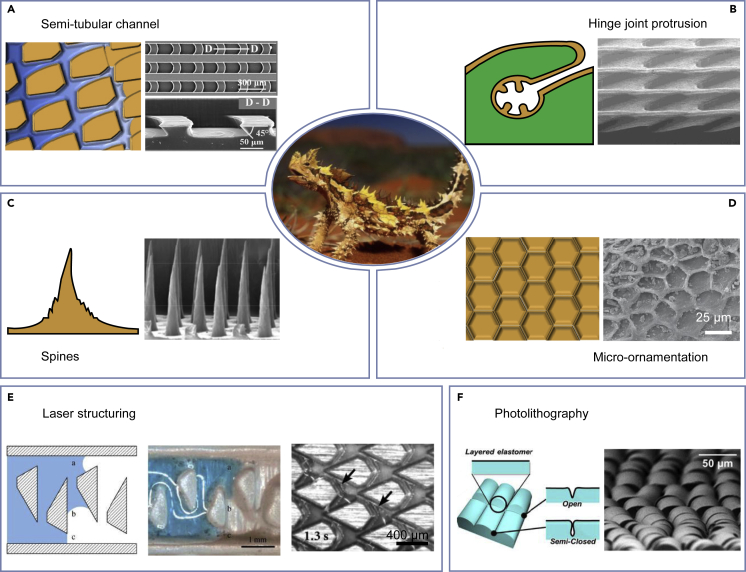
Water management attributes of desert lizards and preparation methods (A–D) Four main water-management characteristics of desert lizard including: (A) Semi-tubular channel. (B) Hinge joint protrusion. (C) Spines. (D) Micro-ornamentation. Adapted with permission from ref.,^139^ copyright 2017 Wiley-VCH; ref.,^140^ copyright 2016 Wiley-VCH; ref.,^66^ copyright 2014 Wiley-VCH; and ref.,^141^ copyright 2021 Wiley-VCH. Middle figure adapted with permission from www.ryanphotographic.com, copyright 2022 Ryan Photographic. (E and F) Preparation processes of existing lizard-inspired water collection devices including: (E) Laser structuring. (F) Photolithography. Adapted with permission from ref.,^46^ copyright 2015 The Royal Society; ref.,^100^ copyright 2016 SPIE; and 141, copyright 2016 American Chemical Society.

Few existing designs focus on learning from lizards. However, based on our previous work on pitcher plants and cactus, we believe there is a broad prospect in learning from the epidermal structure of lizards. The half-open semi-tubular channels existing at the scale-hinge joints of desert lizards were found similar to the peristome surface of the carnivorous plant *N. alata*.^138^ Such biomimetic microgrooves (Figure 6A) have already been fabricated by UV lithography and 3D printing.^7^^,^^48^^,^^139^^,^^140^ These microgrooves can enable liquid to spread at a high speed.^7^ The semi-tubular channel of desert lizards has unique protrusions inside (Figure 6B), which could form narrow subchannels to yield an extension of transport distance.^47^^,^^99^ Rim-shaped protrusions also exists in *N. alata*, which can enhance capillary rise in the transport direction and prevents backflow.^138^ This principle of asymmetrical liquid spreading has fulfilled the biomimetic microgrooves structures an uni-directional liquid transport.^7^^,^^48^ In addition, similar to the spines of cactus, the spines on the epidermis of desert lizards (Figure 6C) can act as condensation foci.^142^ Besides, the micro-ornamentations (Figure 6D) on the surface could increase the surface hydrophilicity.

#### Laser structuring

Inspired by the Texas horned lizard, surface structures have been fabricated for passive, directional transport. As shown in Figure 6E, the principle of directional water transport on *P. cornutum* was simplified to asymmetrical narrowing and specific interconnection of the capillaries.^46^ This identified principle for directional water transport was validated on polymethyl methacrylate (PMMA) by laser structuring, which maintains the directional transport of water.^46^ This mechanism was also transferred to steel by using a pulsed picosecond laser.^100^ Directionality in liquid transport was maintained in the hexagonally arranged channels, The fabricated hexagonally arranged channels can maintain a directional transport of cooling lubricants.

#### Photolithography

Inspired by the epidermis of desert lizards, Cha et al. designed a hybrid structure of fluidic networks via a controlled formation of cracks and folds.^143^ A prepattern was fabricated by photolithography and channel structures were subsequently formed by stress and partial oxidation (Figure 6F). Semi-closed channels were formed by patterning the surfaces with notches and grooves. Due to the interconnected cracks and folds, water could be spontaneously transported into the inner folded channels from the surface cracks via capillary action. Additionally, because of the shape tunability of both the cracks and folds, the fluidic network could be modulated by applying mechanical strain. Such modulation over the fluidic network can be used to control the flow of water.

### Overflow control for water collection

Inspired by natural organisms, various biomimetic water management devices have been constructed. The existing devices mainly capture water by hydrophilic structures or components. Although considerable progress has been made on the hydrophilic surfaces, the water transport and collection speed are limited by the high adhesion and uncontrolled liquid flooding. Additionally, dropwise and film-wise condensation occurs on the hydrophobic low-adhesion and hydrophilic high-adhesion surfaces, respectively.^144^ For the transport and collection of deposited water, the low-adhesion hydrophobic or slippery surfaces are more favorable to avoid accumulation and flooding, enabling a rapid refresh of deposition interfaces to further improve the deposition efficiency.^25^^,^^86^^,^^145^

To address the above-mentioned issue, Feng et al. fabricated hydrophobic inclined Janus pillar arrays with height gradient.^146^ Directional transport of condensed droplets from the flat to the curved side can be induced on the tip of the Janus pillar. Therefore, condensed droplets moved and merged spontaneously to achieve the directional, continuous, and ultrafast transport of water. Another example of directional droplet transport on hydrophobic surfaces was the drain fly tentacle which has parabola-shaped knots forming a series of inclined ratchet arrays with gradient tilt angle.^147^ The condensate droplets growing in a single ratchet were gradually driven toward the tip by the capillary force in the tilted ratchet direction and then merge with the neighboring droplets. After repeated propel and coalescence, large droplets can be collected at the tip of the tentacle.

Rapid nucleation and efficient transport are often contradictory. Bioinspired slippery surfaces have been proposed as a tool to improve both nucleation and transport, so as to improve fog collection efficiency.^86^^,^^145^^,^^148^^,^^149^ Park et al. proposed a multi-bioinspired approach and developed a surface structure with slippery asymmetric bump arrays.^86^ This model incorporates the *Namib Desert* beetle’s efficient nucleation, cactus’s directional transport, and pitcher plant-inspired slipperiness. Water nucleation and transport were simultaneously improved. Li et al. proposed a fog collection mechanism with slippery aqueous layer inspired by the pitcher plant.^7^ Compared with the dry surface, the water droplets can achieve ultrafast sliding on the surface wetted by the precursor water film. On the wet and slippery surface, the water transportation speed was increased by 300 times, and the fog collection efficiency was increased by 5 times. A multifunctional biomimetic device was developed to collect not only water but also oil and organic fog, enabling different practical application scenarios such as water collection in the cooling tower and organic fog collection in chemical plants, laboratories, and kitchens.

Learning from pitcher plants and rice leaves, Dai et al. fabricated a biomimetic surface consisting of nanotextured directional microgrooves in which the nanotextures are infused with hydrophilic lubricant.^145^ Under the synergistic effect, water droplets nucleated rapidly and coalesced effectively. The fog collection efficiency results to be much higher than that of hydrophobic or nondirectional slippery surfaces. Inspired by the directional pumping strategy of emergent aquatic plants, Zhang et al. developed a lubricant-infused slippery surface with hollow hydrogel bump arrays.^149^ Based on asymmetric capillary forces of the curved slippery liquid surface lifted by the hydrogel bum, nucleated water droplets from all directions can be attracted and collected to the hollow bumps.

From structured surfaces to bioinspired slippery surfaces, unprecedented droplet growth and transport were achieved, with enhanced water collection efficiency. In the above examples, the curvature structure of the surface affects the liquid deposition to a certain extent. Additionally, the inherent chemical compositional hydrophilicity also has a significant effect on droplet deposition. Besides, several developed biomimetic structures such as cones and arrayed structures such as pitcher plant peristome-mimetic arrays utilize their capillary force to transport the deposited liquid directionally for efficient and controllable collection.^7^^,^^16^ Therefore, rational design of surface chemical composition and multi-curvature structure can simultaneously improve droplet deposition and transport, eventually improving water collection efficiency.

### Conclusion and outlook

The imbalance between demanding and available freshwater is worsening in recent years due to environmental pollution, population growth, and economic development. Deserts are the driest places in the world and desert creatures all over the world have evolved special adaptations to survive in this extreme water shortage environment. In this review, the mechanisms of water harvesting mechanism from the fog were summarized, and how they were utilized by the four typical desert creatures, cactus, desert beetles, lizards, and snakes, to adapt their living desert type was elaborated. The recent progress in the manufacturing methods of the biomimetic water-collecting structures and the influence of overflow on water collection were demonstrated.

**Figure 7:**
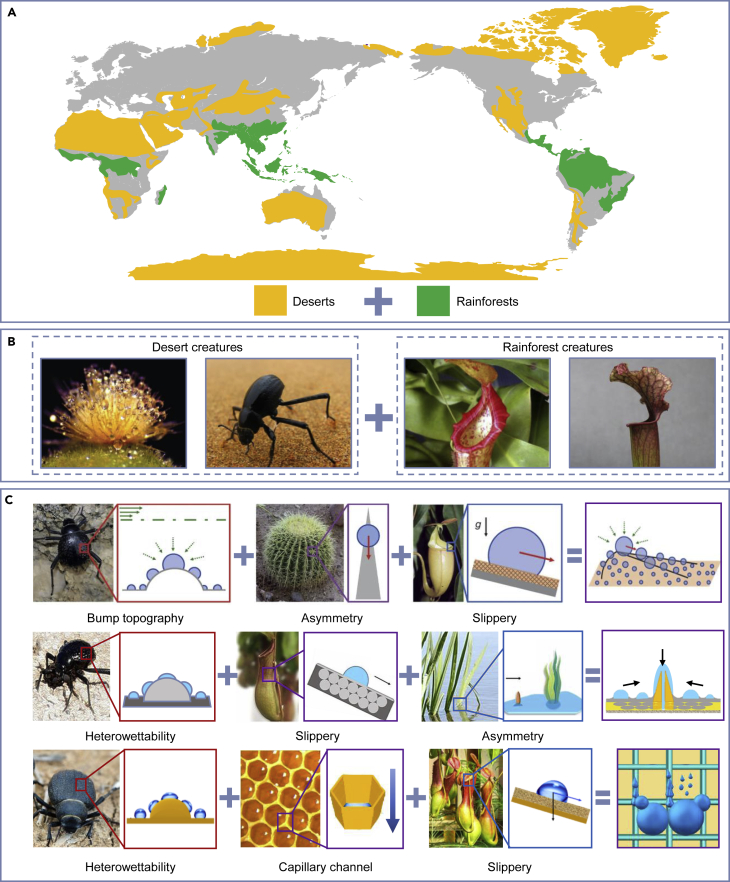
Geographical distribution of extreme environments and the extreme dweller-inspired systems (A) Geographical distribution of the two extreme conditions, including the deserts and the rainforests. (B) Potential to incorporate attributes of creatures from desert and rainforest to increase efficiency and enable new application. Adapted with permission from ref.,^15^ copyright 2014 American Chemical Society; www.flickr.com, copyright James Anderson; ref.,^7^ copyright 2020 PNAS; and ref.,^6^ copyright 2018 Springer Nature. (C) Examples that utilizes different characteristic including hetero-wettability bumps in *Namib Desert* beetles, asymmetric spine structures in cactus, slipperiness of *Nepenthes* pitcher plants, pumping strategy of emergent aquatic plants, and capillary channel of honeycomb network. Adapted with permission from ref.,^86^ copyright 2016 Springer Nature; ref.,^149^ copyright 2019 PNAS; and ref.,^135^ copyright 2021 American Chemical Society.

Although great progress has been made in developing bioinspired water management devices, the current designs have remaining shortages. While extremely dry and harsh environments in the desert have evolved special adaptations in the desert creatures to survive water-shortage conditions, these characteristics may not be applicable in other places and in various application scenarios. Another part of the ecosystem, the tropical rainforest, have extremely high temperature and high humidity. Their soil can be regarded as “poor” since the lack of nutrients, resulting from the rapid decomposition of litter and heavy, frequent rains which wash the minerals of the soil out.^150^ Similar to desert creatures, rainforest creatures have developed special adaptations to these surroundings. Examples include: the pitcher plant *N. alata* which enables a continuous, directional, water transport on its highly modified peristome surface; the *Sarracenia* operculum which fulfills ultrafast water harvesting and transport.^6^^,^^138^ These natural wisdoms can separately help the creatures to survive in their desert or rainforest habitat. When designing bioinspired devices, it is reasonable to incorporate those key characteristics in creatures from different habitats, aiming at increasing efficiency and allowing potential applications (Figure 7A and 7B).

As shown in Figure 7C, several examples of multiple-inspired designs are presented. Park et al. proposed a multi-bioinspired approach and developed a surface structure with slippery asymmetric bump arrays.^86^ This model incorporates the *Namib Desert* beetle’s efficient nucleation on bump topography, cactus’s directional transport on asymmetric spine, and pitcher plant-inspired slipperiness. This rational design strategy can enable a wide range of water-harvesting and phase-change heat-transfer applications. Zhang et al. developed a lubricant-infused slippery surface with hollow hydrogel bump arrays.^149^ This model was inspired by the hetero-wettability bumps in *Namib Desert* beetles, the slipperiness of *N. alata* peristome surface, as well as the pumping strategy of emergent aquatic plants. Based on asymmetric capillary forces of the curved slippery liquid surface lifted by the hydrogel bump, this model can fulfill applications including droplet capturing, pumping, and collecting. Zhang et al. presented a bioinspired patterned fog collector with hydrophilic nanofibrous bumps and a hydrophobic slippery substrate for spontaneous and efficient fog collection.^135^ This design incorporates the hetero-wettability bumps in *Namib Desert* beetles, honeycomb network-inspired nanofibrous capillary channel, and pitcher plant-inspired slippery substrate. The hydrophilic nanofibrous bumps increase the effective fog-collecting area, while the hydrophobic slippery substrate promotes rapid transport of collected water, finally achieving high-efficiency water collection.

Efforts should be paid into incorporating different creatures and finding more species that have gifts in handling water with special structures. There are already many species without thorough investigation to show superior water collection efficiency and enable applications other than fog collection. Desert lizards can be an excellent example, which utilize their skin to transport water from multiple sources to their mouth.^26^ To learn from desert lizards and incorporate the merits of other species, potential applications including antigravity water transport and spontaneous agricultural irrigation can be fulfilled. Some other species with even better water management efficiency may still remain unknown.

As discussed above, combining the merits of various creatures into an integrated system could further increase the performance of water management devices. The increasingly severe shortage of freshwater resources will promote further research on bioinspired water management strategies. Further research will continue to improve the design and narrow the gap between basic research and applied technology. In no time, bioinspired water management devices with highly efficient and low cost will become a key component of human sustainable development.
